# The Impact of High-Intensity Ultrasound-Assisted Extraction on the Structural and Functional Properties of Hempseed Protein Isolate (HPI)

**DOI:** 10.3390/foods12020348

**Published:** 2023-01-11

**Authors:** Shunyu Yao, Wu Li, Yue Wu, Gregory J. O. Martin, Muthupandian Ashokkumar

**Affiliations:** 1 Sonochemistry Group, School of Chemistry, The University of Melbourne, Parkville, VIC 3010, Australia; 2 Algal Processing Group, Department of Chemical Engineering, The University of Melbourne, Parkville, VIC 3010, Australia

**Keywords:** ultrasound, hempseed protein isolates, free sulfhydryl content, protein structure modification, functionality

## Abstract

Hempseed protein has become a promising candidate as a future alternative protein source due to its high nutritional value. In the current study, hempseed protein isolate (HPI) was obtained using ultrasonic-assisted extraction with the aim to improve the functionality of HPI via protein structure modification. The solubility of HPI could be improved twofold under 20 kHz ultrasound processing compared to conventional alkaline extraction-isoelectric point precipitation. The protein solubility was gradually enhanced as the ultrasonic power improved, whereas excessive ultrasound intensity would cause a decline in protein solubility. Ultrasonic processing was found to have beneficial effects on the other functionalities of the extracted HPI, such as emulsifying and foaming properties. This improvement can be ascribed to the physical effects of acoustic cavitation that changed the secondary and tertiary structures of the protein to enhance surface hydrophobicity and decrease the particle size of the extracted protein aggregates. In addition, more available thiols were observed in US-treated samples, which could be another reason for improved functionality. However, the results of this study also revealed that prolonged high-power ultrasound exposure may eventually have a detrimental impact on HPI functional properties due to protein aggregation. Overall, this study suggests that high intensity ultrasound can enhance the functionality of HPI, which may ultimately improve its value in HPI-based food products.

## 1. Introduction

The increasing global population requires new and sustainable sources of dietary protein that can help address the insufficiency of conventional protein sources. Hempseed has always been regarded as a by-product from industrial oil and fiber extraction. However, whether it has potential to provide high-quality edible oil and dietary protein for the food industry remains underdeveloped [1]. Among all hempseed-based products, hempseed protein is attracting attention due to its beneficial amino acid composition and excellent digestibility [2,3]. Edestin is the major storage protein of hempseed protein composition, which consists of two types, CsEde1 and CsEde2 [4]. CsEde2, especially, exhibited abundant cysteine (1.07%) residue, which contains high sulfhydryl content. The current HPI extraction protocol conditions include a high pH (9–12), high temperature (32–80 °C), and long extraction time (1–4 h) to achieve viable protein yields [5,6,7,8]. However, these conditions also provide an excellent environment for thiol deprotonation followed by intermolecular disulfide bond formation in the HPI product. To the best of our knowledge, no literature has reported the dominance of thiol content to protein functionality in the hempseed system.

To improve the functionality of HPI, various structural modification methods have been conducted, such as heating [8], succinylation [9], and pH-shifting [10]. However, all of these approaches are not suitable for food processing, due to the addition of toxic chemicals, nutrition loss, and less sensory acceptance. Moreover, most of the previous studies still focused on the macro mixing scale (magnetic stirring) for hempseed protein extraction. There is still a lack of literature on the micro mixing scale in the hempseed extraction system. Therefore, it is preferable to find a novel practical technique to enhance the functionality of HPI in order to broaden its usefulness.

The impact of high-intensity ultrasound (HIU) on plant protein extraction has been widely investigated for decades [11]. HIU can generate a series of physical effects via acoustic cavitation, such as high shear force, micro-jets, turbulent flow, and high temperature and pressure in localized areas. Many researchers have studied HIU (ultrasound intensity >1 W/cm^2^) under various frequencies (20–100 kHz) to improve the protein extraction process [12,13]. In general, low frequency ultrasound (20 kHz) is found to be useful in food processing due to the generation of strong physical forces. Ultrasound has been reported to be an effective technique in protein aggregation prevention. Zisu et al. [14] reported that ultrasound could significantly hinder the extent of protein aggregation in the whey protein system, as indicated by the increase in the clarity of WPC solutions. Moreover, Mohapatra et al. [15] discovered that ultrasound is able to control polymer chain growth and yield a narrower PDI in US-induced polymerization. On the other hand, recent studies have demonstrated that HIU can also improve plant protein solubility and functional properties by causing protein structure changes [16,17]. Hu et al. [18] discovered that the application of excessive US power might result in protein aggregation in the soy protein system. In another investigation, Jiang et al. [19] also reported that longer sonication time and excessive US power resulted in poor protein solubility in black bean protein isolate system. However, there is a limited number of investigations regarding the effect of ultrasound in HPI systems. Karabulut et al. [20] investigated the optimization of ultrasound conditions on the protein extraction yield and its functionality. In another investigation, Liu et al. [21] studied the effect of various ultrasound conditions on improving physical properties. None of them have considered the impact of disulfide bond formation during the extraction in the HPI system. According to the literature, the reduced functionality most likely occurs at the alkaline extraction step. Despite this, the feasibility of ultrasound-assisted alkaline extraction has not been studied yet. Therefore, hempseed contains unique proteins, and there is still a lack of systematic investigation regarding the connection of thiol chemical reactivity to its functionality.

Therefore, this study aims to systematically analyze different ultrasound-assisted extraction parameters on hempseed protein molecular property changes during and after alkaline extraction. To achieve a good balance between protein yield and protein functionality, suitable ultrasound parameters were first determined by preliminary tests. Then, the functionality of US-modified HPI products was compared to that of HPI obtained based using the conventional AE-IEP method. Ultimately, the mechanism of ultrasound-assisted hempseed protein extraction is proposed, which is completely missing in the literature. This study may provide new insights into a fundamental understanding of the effect of ultrasound on the extraction yield and properties of HPI. 

## 2. Materials and Methods

### 2.1. Materials

Non-dehulled commercial hempseed powder (supplied by Maxarham) was utilized as the hempseed protein source in this study. All the chemicals and reagents were of analytical grade purchased from Sigma-Aldrich and stored at room temperature (24 °C) until use. Criterion™ Precast Midi PAGE Gels were obtained from Bio-Rad (Bio-Rad Technologies, Gladesville, Australia) and stored at 4 °C until use. Protein assay dye reagent concentrate (Catalog#500-0006) was obtained from Bio-Rad. Canola oil was purchased from Woolworths, Australia. Mille-Q water (Millipore Australia Pty Ltd., NSW, Australia) was used in all experiments with a typical resistivity of 18.2 MΩ-cm at 24 °C.

### 2.2. Preparation of HPI Based on AE-IEP and Ultrasonic-Assisted Extraction

HPI was prepared based on Hadnađev’s method, with a slight modification [22]. A mass of 250 g hempseed powder was first dispersed in 500 mL of hexane at room temperature for 16 h to remove the excessive lipids and eliminate the impact of the lipids in the protein characterization. Then, the dispersion was centrifuged at 4383× *g* (Megafuge 8, Thermo Fisher Scientific Inc., Waltham, MA, USA) for 10 min to remove the solvent. The pellet was then re-dispersed in hexane for another defatting cycle, and this process was repeated at least two times until the supernatant was colorless. Then, the pellet was collected and placed in an oven (set at 60 °C) overnight to ensure the hexane was entirely removed. The defatted hempseed powder was mixed with distilled water with a weight/volume ratio = 1:15, and the pH was adjusted to 10.0 using 1 M NaOH. The extraction process was conducted at 37 °C and stirred using a magnetic stirrer for 2 h. The dispersions were then centrifuged at 4383× *g* for 20 min. The bottom layer was discarded, the supernatant was collected and vacuum-filtered, and the filtrate was subjected to acidic precipitation at pH = 4.5 with 1 M HCl. The suspension was centrifuged at 4383× *g* for 20 min, and the precipitate was collected and washed using distilled water for at least four cycles to remove excess salt. The de-salting precipitate was then re-dispersed in deionized water, and the pH was adjusted to 7.0 using 1 M NaOH and lyophilized for 72 h to obtain the HPI powder.

For HPI obtained via ultrasound-assisted extraction, only the alkaline extraction step was slightly modified. The defatted hempseed powder was mixed with distilled water with a weight/volume ratio = 1:15, and the pH was adjusted to 10.0 using 1 M NaOH. Afterwards, the dispersions were subjected to ultrasonic treatment using a high-intensity ultrasound horn (20 kHz, Branson 450 digital sonifier, Marshall Scientific Inc., Hampton, NH, USA) with 0.6 cm diameter. The bottom of the horn was placed at the center of the suspensions for equal distribution of power. An ice bath was used during the ultrasound to avoid overheating the protein. 

### 2.3. Mechanistic Investigation on US-Assisted Hempseed Extraction

For mechanistic study purposes, the extracts were collected in centrifuge tubes at 1, 3, 5, 7, and 10-min during US-assisted extraction (under 11.2, 26.8, and 50.0 W/cm^2^). The solutions were centrifuged at 4383× *g* for 20 min (Megafuge 8, Thermo Fisher Scientific Inc., Waltham, MA, USA). The fresh supernatant was carefully collected for further characterization (the determination of protein concentration and free -SH content). This work was aimed at monitoring the hempseed protein extraction kinetic, which could auxiliary prove our proposed mechanism.

### 2.4. Ultrasound Calorimetry Power and Intensity Determination

Calorimetric investigations were conducted by sonicating 100 mL of water for specified time intervals and recording the temperature changes. Acoustic power and energy intensity were then calculated based on Equations (1) and (2) [23]:

(1) P=m*Cp*(dTdt)

(2) Ia=PA 

where *P* (W) is the ultrasonic calorimetric power, *m* is the mass of sonicated water (*m* = 0.1 kg), *C_p_* represents the specific heat capacity of the sonicated medium (*C_p_* of water = 4.2 × 10^3^ J/kg·K), and *dT/dt* represents the slope of temperature increase versus specific time intervals. *I_a_* represents ultrasound power intensity (W/cm^2^), and *A* is the surface area (cm^2^) of the ultrasound horn.

### 2.5. Determination of Protein Concentration (Protein Assay)

In this study, the protein concentration was determined using a BCA protein assay kit (Bio-Rad Technologies, Gladesville, Australia) [24]. Briefly, 10 μL of protein samples were injected into separate microtiter plate wells (96 wells). Then, 200 μL of diluted dye (1:4 dilution) was injected into each well. The sample and assay dye were thoroughly mixed using a microplate mixer (Spectro star Nano, BMG Labtech, Australia) for 5 min. The absorbance was read at 595 nm using an absorbance microplate reader (Spectro star Nano, BMG Labtech, Australia). A standard curve was first established by the bovine serum albumin (BSA) standard, and the unknown protein concentration could be subsequently calculated from the regression equation. At least three replicates were conducted for each measured sample. BCA assay could provide the protein yield changes during the extraction. Furthermore, all the experiments conducted in this work were standardized to the same initial protein concentration based on the current assay.

### 2.6. Determination of Free -SH Group Content

The free -SH group content of the soluble protein samples was determined using 5,5-dithio-bis-(2-nitrobenzoic acid) (DTNB) based on the DTNB assay method reported by Beveridge et al. [25] with slight modification. The free -SH content is crucial in the current research. Owing to the intermolecular SS bond formation might occur during the extraction, which could consume the available free sulfhydryl content. Hence, the DTNB-detectable sulfhydryl content changes could indirectly reflect the extent of disulfide bond formation. The protein samples were dispersed in Tris-Gly standard buffer solution (86 mM Tris, 90 mM glycine, and 4 mM EDTA, pH 8.0). The mixtures were centrifuged at 4383× *g* for 20 min, and the supernatants were first collected for protein concentration determination. Afterwards, the supernatants were diluted using Tris-Gly buffer, aiming to have 0.2% protein concentration (2 mg/mL). For free -SH determination, 40 μL of DTNB (4 mg/mL) solution was added into a 4 mL aliquot of the protein supernatant. The mixture was shaken and incubated at 24 °C for 15 min and subjected to spectrometric analysis (Cary 3E UV-vis spectrophotometer, Varian, Australia). The absorbance was read at 412 nm, and Tris-Gly buffer was used as a reagent blank. The free -SH content in the soluble fraction of protein can be calculated as Equation (3) [26]:

(3) $$SH\;\left(\mu \mathrm{mol}/g\;soluble\;protein\right)=\frac{10^{6}\times A\times D}{1.36\times 10^{4}\times c}$$

where *A* is the absorbance, *D* represents the dilution factor, 1.36 × 10^4^ M^−^^1^cm^−^^1^ is the molar extinction coefficient, and c is the initial protein concentration (2 mg/mL) used in this study.

### 2.7. Amino Acid Composition (HPLC)

It has been reported that ultrasound may affect the primary structure of proteins by oxidizing the amino acid residues. Therefore, the effect of ultrasound on the amino acid composition of HPI was investigated following the method reported by Wu et al. [27]. Briefly, untreated or sonicated HPI solution (2 mg/mL) was mixed with 6 M HCl at a ratio of 1:1 (*v*:*v*) and was then transferred into a 1 mL Thermo Scientific™ vacuum hydrolysis tube (Thermo Fisher Scientific Inc., Waltham, MA, USA). Next, the tube was slowly sealed at a vacuum pressure of 0.8 bar using a vacuum pump. The sealed tube was placed into a metal block and heated at 110 °C for 20 h to hydrolyze the HPI into amino acids. Finally, hydrolyzed samples were filtered with a 0.45 μm filter and stored at 4 °C before analysis.

An Agilent HPLC system (1260 Infinity II, Agilent Technologies Pty Ltd., Santa Clara, CA, USA) coupled with a fluorescence detector (FLD) was applied to quantify the amino acids. The stationary phase consists of an Agilent RP Poroshell C-18 column (100 × 4.6 mm, 2.7 μm), and the mobile phase was composed of phase A and phase B. Mobile phase A contained 10 mM Na_2_HPO_4_, 10 mM Na_2_B_4_O_7_ and 5 mM NaN_3_ with pH 8.2. Mobile phase B contained HPLC-grade acetonitrile, HPLC-grade methanol, and deionized water at a ratio of 45:45:10 (*v*:*v*:*v*). The gradient program was set as follows: 0–0.35 min, 2% B; 0.35–13.4 min, 57% B; 13.4–13.5 min, 100% B; 13.5–15.7 min, 100% B; 15.7–15.8 min, 2% B; 15.8–18 min, 2% B. The excitation and emission wavelength were set at 230 nm and 460 nm, respectively. The column temperature was 40 °C, and the flow rate was 1.5 mL/min. To acquire the spectra of primary amino acids, the online derivatization of amino acids was performed. A total of 0.4 mL of H_3_PO_4_ was added into 100 mL of mobile phase A as the injection diluent, and other reagents (borate buffer, OPA and FMOC) were all bought from Agilent Technologies Pty Ltd. The injection program is summarized in Table 1. The concentration of each amino acid was calculated based on the calibration curves of amino acid standards and expressed as mM.

### 2.8. Circular Dichroism (CD)

Ultrasound can affect the secondary structure of proteins to change their physicochemical properties and functionality [28]. Therefore, the secondary structures (α-helix, β-sheet, β-turn, and random coil) of untreated and sonicated HPI were measured with a CD spectrometer (Chirascan plus, Applied photophysics, Skipton, UK). HPI solution was diluted with deionized water to 0.1 mg/mL. Then, it was transferred into a quartz cell with a 1-mm path length. The wavelength was scanned from 260 nm to 190 nm with a bandwidth of 1 nm, and each spectrogram was an average of three spectra accumulations. The secondary structure was analyzed by the CD tool software.

### 2.9. Intrinsic Fluorescence Emission

Emission fluorescence spectroscopy was used to identify microenvironment changes of tyrosine (Tyr) and tryptophan (Trp) residues, which are related to HPI tertiary structure [29]. Original and sonicated HPI solutions were diluted with deionized water to reach a concentration of 0.1 mg/mL, and all spectra were obtained using an Agilent Cary Eclipse fluorescence spectrophotometer (Agilent Technologies Pty Ltd., Santa Clara, CA, USA). In brief, 3 mL of diluted HPI solution was added to a quartz cuvette with a 1 cm path length. The excitation wavelength was set at 280 nm, and the emission wavelength was measured from 290 nm to 450 nm. The slit widths of emission and excitation were both 5 nm, and the photomultiplier tube (PTM) voltage was set to ‘medium’ for all measurements. The measurements were performed in duplicate.

### 2.10. Identification of Protein Subunits (SDS-PAGE)

Reducing and non-reducing SDS-PAGE can be used to identify the change in the molecular weight of HPI and the formation of intermolecular disulfide bonds [30]. Different sample buffer solutions were prepared first. For non-reducing SDS-PAGE, Laemmli sample buffer (Bio-Rad Laboratories Pty., Ltd., South Granville, Australia) was applied directly to disrupt the quaternary, tertiary, and secondary structure and maintain covalent and disulfide bonds. For reducing SDS-PAGE, 50 μL of β-mercaptoethanol was added into 950 μL of Laemmli sample buffer solution as the sample buffer to break all intramolecular and intermolecular disulfide bonds. In this study, the non-reducing SDS-PAGE was our main focus, as it can reflect the extent of protein oligomer formation. In addition, the result could provide support to our proposed mechanism. Subsequently, untreated or sonicated HPI solutions of 2 mg/mL were diluted with non-reducing sample buffer (Laemmli sample buffer) or reducing sample buffer (the mixture of Laemmli sample buffer and β-mercaptoethanol) at a ratio of 1:1 (*v*:*v*). Then, diluted samples were heated at 90 °C for 5 min to linearize protein structures following a cooling at room temperature. Tris/SDS running buffer (Bio-Rad Laboratories Pty., Ltd., South Granville, Australia) was diluted with deionized water at a ratio of 1:9 (*v*:*v*). The integrated upper buffer chamber and lower buffer tank were filled with approximately 60 mL and 400 mL of diluted running buffer. Afterwards, 30 μL of cooled samples and protein standard (Bio-Rad Laboratories Pty., Ltd., Australia) were added into different gel lanes. The gel was run at a constant voltage of 200 V for 40 min and then washed with deionized water. A Bio-Safe™ Coomassie Stain (Bio-Rad Laboratories Pty., Ltd., Australia) was applied to stain the gel for 1 h with gentle shaking, after which the gel was de-stained with deionized water to image using a Gel Doc™ XR (Bio-Rad Laboratories Pty., Ltd., Australia).

### 2.11. Surface Hydrophobicity (H_0_)

Protein surface hydrophobicity was determined using 1-anilinonaphthalene-8-sulfonate (ANS) as a fluorometric probe [31]. The HPI solutions were diluted with PBS buffer (20 mM, pH 7.0) to have at least four different concentrations between 0.05~0.5 mg/mL. Afterwards, 20 μL of ANS (8 mM) was added to 4 mL protein solutions. The protein solutions were agitated and incubated at room temperature in the dark for 10 min. The fluorescence intensity was recorded using an excitation wavelength of 390 nm and the emission wavelength of 470 nm with a fluorescence spectrophotometer (Agilent Technologies, Inc., Santa Clara, CA, USA). The slit widths of emission and excitation were both 5 nm, and the photomultiplier tube (PTM) voltage was set to ‘medium’ for all measurements. The surface hydrophobicity was calculated as the slope of the linear regression equation (fluorescence intensity as a function of protein concentration).

### 2.12. Protein Percentage Solubility

The percentage protein solubility of HPI samples was determined according to the method described by Malomo et al. [32] with some modifications. Briefly, 20 mg of HPI powder was dissolved in 10 mL phosphate-buffered saline (PBS buffer, 20 mM) at a pH 5.0 (HPI isoelectric point), 7.0 (neutral pH), and 14.0 (considered as a fully dissolved pH point). The mixtures were subjected to orbital shaking at 24 °C overnight, followed by centrifugation at 4383× *g* for 10 min. The supernatant was collected and used to determine the protein concentration by the same BCA assay shown in Section 2.4. Hence, the percentage protein solubility (*PS* (%)) was calculated using Equation (4):

(4) $$PS\;\left(\%\right)=\frac{C_{n}}{C}\times 100$$

where *C_n_* represents the concentration of the soluble fraction in HPI at pH value *n* (*n* = 5.0, 7.0), and *C* stands for the concentration of the soluble fraction in HPI at pH value at pH 14.

### 2.13. Protein Emulsifying Properties

The emulsifying ability index (EAI) and the emulsifying stability index (ESI) were determined based on the methods described by Guo et al. [33] with minor modifications. 

Firstly, emulsions were formulated at a standardized protein concentration (0.25 wt%, pH 7.0) with 5 wt% canola oils as the dispersed phase. The aqueous and oil phases were homogenized in a plastic container with a total volume of 18.76 mL and mixed using an Ultra-Turrax (IKA-WERKE, T25 Basic) high-shear mixer at 13,500 rpm for 5 min. After mixing, a fixed volume of 20 μL of the emulsion was taken from the tube bottom and immediately diluted with 5 mL SDS solution (0.1 wt%). Subsequently, the absorbance of the emulsion was read at 500 nm using a UV-Vis spectrometer (Cary 50 bio UV-Vis, Agilent, Mulgrave, Australia). *ESI* was determined by examining the sample absorbance after 10 min. The *EAI* and *ESI* were calculated using Equations (5) and (6):

(5) $$EAI\;\left(m^{2}/g\right)=\frac{2\times 2.303\times DF\times A_{0}}{c\times l\times \left(1-\emptyset \right)\times 10^{4}}$$

(6) $$ESI\;\left(min\right)=\frac{A_{0}}{A_{0}-A_{10}}\times t$$

where *DF* stands for the dilution factor, c denotes the initial protein concentration (g/mL), *l* is the optical path (*l* = 0.01 m) and ∅ represents the emulsion’s oil proportion (*v*/*v*), and *A*_0_ and *A*_10_ are the emulsion absorbance at 0 and 10 min.

### 2.14. Particle Size and Microstructure

A light scattering instrument (Mastersizer 3000, Malvern, UK) was used to measure the volume-weight mean size *D*_4,3_ ($D_{4,3}=\frac{\sum n_{i}r_{i}^{4}}{\sum n_{i}r_{i}^{3}}$), where *n_i_* and *r_i_* are the droplet number and droplet radius) [34]. The particle size of HPI could reflect the protein aggregation difference in the ultrasound-assisted and control samples. Moreover, the emulsion droplet size difference could reflect the emulsifying ability in different samples. The HPI samples were first transferred into 5 mL distilled water, aimed to have a 1.0 wt% initial concentration. Subsequently, a suitable number of drops from HPI suspensions were dripped into the measurement beaker of the device. The droplet size was measured using laser diffraction analysis, and the droplet size and size distribution were determined by the Malvern Mastersizer software.

An optical microscope was used to further support our findings relating to the emulsifying properties. Emulsion microstructure was recorded using an optical microscope (Olympus Model IX7, Melbourne, Australia) to ensure emulsion homogeneity. For the measurement, 10 µL of the emulsions was loaded on the microscope slide, and the images were captured using an inverted 60× objective lens (Olympus, UPlanFL, Tokyo, Japan). The images were taken for each emulsion sample from three individual experiments.

### 2.15. Foaming Properties

Foaming capacity (FC) and stability (FS) properties were determined based on the method reported by Li et al. [35] with some modifications. For foam formation, 20 mL of HPI protein solutions (2.0 wt%) diluted using distilled water were homogenized at 13,500 rpm for 2 min using an Ultra-Turrax (IKA-WERKE, T25 Basic) high-shear mixer. The suspensions were immediately transferred into a 100 mL measuring cylinder, and the total volume was recorded as *V*_0_. The mixtures were allowed to stand for 20 min at room temperature, and the volume was recorded again (*V*_20_) to determine the protein foaming stability. Then, the *FC* and *FS* were calculated by Equations (7) and (8):

(7) $$FC\;\left(\%\right)=\frac{\left(V_{0}-V_{I}\right)}{V_{I}}\times 100$$

(8) $$FS\;\left(\%\right)=\frac{\left(V_{20}-V_{I}\right)}{V_{0}-V_{I}}\times 100$$

where *V*_0_ is the total volume after homogenization at 0 min, *V*_20_ represents the total volume after keeping at room temperature for 20 min, and *V_I_* stands for the initial volume of the suspension (*V_I_* = 20 mL).

### 2.16. Water Holding Capacity and Oil Holding Capacity

Water (WHC) and oil holding capacity (OHC) were determined according to the method described by Stone et al. [36] with minor modifications. HPI (1.0 g) was suspended with either deionized water (10.0 g, pH 6.8) or canola oil (10.0 g) in a 50 mL centrifuge tube. The tubes were completely vortexed by a shaking table and incubated at 24 °C for 30 min. Afterwards, samples were centrifuged at 4383× *g* for 20 min. The supernatant was cautiously decanted, and the tube was inverted for another 30 min to drain the remaining supernatant. Subsequently, the pellet was weighed for *WHC* and *OHC* calculation. *WHC* and *OHC* were determined by Equations (9) and (10):

(9) $$WHC\;\left(\%\right)=\frac{m_{w}-m_{I}}{m_{I}}\times 100$$

(10) $$OHC\;\left(\%\right)=\frac{m_{O}-m_{I}}{m_{I}}\times 100$$

where *m_w_* stands for the total weight of the tube after HPI absorbed the water, *m_o_* represents the total weight of the tube after HPI absorbed the oil, and *m_I_* is the initial weight of the tube content (the mass of the tube plus the initial weight of HPI).

### 2.17. Statistical Analysis

All experiments were repeated three times, and measurements were performed on the triplicate samples. Data were expressed as mean ± standard derivations and analyzed by analysis of variance (ANOVA). The significance of the differences between variables was found by ANOVA analysis and was defined at the *p* < 0.05 level.

## 3. Results

### 3.1. Mechanistic Investigation of US-Assisted Hempseed Protein Extraction

As aforementioned, two chemical reactions might occur simultaneously during hempseed protein extraction. Namely, protein solubilization and disulfide bond formation. With the purpose of whether US promotes or inhibits protein extraction and thiol reactivity, we have carried out the investigations of protein yield and free thiol content as a function of extraction time.

**Figure 1:**
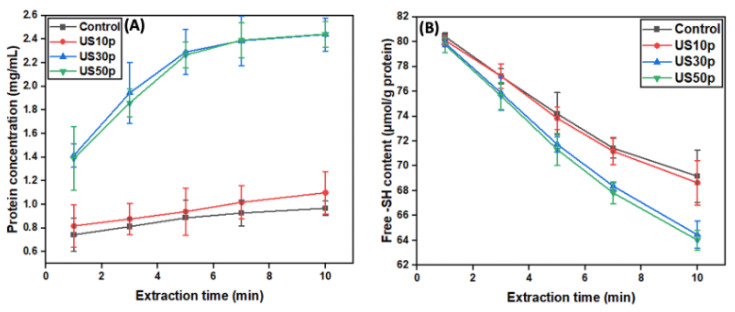
The diagram of the effects of ultrasound as a time function on the hempseed protein extraction process. The protein concentration and free sulfhydryl content changes could reflect the process of protein solubilization and disulfide bond formation. The alkaline extraction condition was set at a fixed pH of 10.0. (**A**) The effect of ultrasound power-time on protein extraction yield. (**B**) The effect of ultrasound power-time on thiol content changes. In this figure, control represents using the conventional magnetic stirring method. US10p, US30p, and US50p represent the sonication power of 11.2 W/cm^2^, 26.8 W/cm^2^, and 50.0 W/cm^2^, respectively. Experiments were conducted in triplicates.

The conventional AE-IEP extraction protocol used magnetic stirring, which focused on the macro mixing scale, as the cavitational effects generated by ultrasound can accelerate chemical reactions by enhancing mass transfer at microscale. As shown in Figure 1A, at a certain extraction time interval, the extraction yields slightly increased under 11.2 W/cm^2^ power density. Strikingly, the extraction yield can increase ~twofold as the ultrasound power was increased. Physical effects, such as turbulent flow and liquid circulation generated by ultrasound, bring about this significant improvement in mass transfer in the system. Interestingly, the extraction yield changes at 50.0 W/cm^2^ were negligible in comparison to that of 26.8 W/cm^2^. This could be ascribed to the high possibility of protein re-aggregation or degradation under higher US power. Therefore, ultrasound power density of 26.8 W/cm^2^ will be first selected for the optimized sonication time assessment.

In the literature, researchers have suggested a few reasons for the poor functionality of HPI after AE-IEP extraction. The hempseed protein nature and extraction conditions might lead to intermolecular SS formation in the system. Therefore, thiol content changes in the HPI extraction system are essential for protein early functionality evaluation. Unsurprisingly, it can be seen in Figure 1B that the trend is similar to protein extraction yield. Long extraction time and high US power lead to a greater decline in thiol content, indicating more edestin oligomers generated in the system. In this study, we evaluated the free thiol changes of the extracted supernatant. Almost all proteins can be solubilized at the alkaline extraction stage; at the same time, edestin monomers link via intermolecular disulfide bonds. Notably, the peptide chains would not fold until hydrogen ions (H^+^) were introduced to the system. Owing to the current protein structure being fully DTNB-accessible (no -SH content was buried in the interior), the figure of the thiol content is roughly equal to the total thiol content. Further research is required to be conducted for protein functionality to assess whether disulfide bond formation would cause detrimental effects on the HPI products.

### 3.2. The Optimization of Ultrasound Parameter Selection

This study aims to determine an optimal sonication time to obtain a balance between HPI yield and its functionality. We selected 1-, 5-, and 10-min extraction time based on the preliminary investigations for US-assisted extraction optimization.

Based on the literature, most hempseed protein extraction was based on the AE-IEP extraction method. However, the highest yield obtained was only 24.2 ± 0.2% by Hadnađev et al. [22], with the low yield possibly due to the mass transfer limitations when using magnetic stirring. Based on ultrasound-assisted extraction, ultrasound can significantly improve the mass transfer in the system by acoustic cavitation, therefore accelerating the extraction process. As shown in Table 2, the extraction yield of HPI was indeed enhanced by ultrasound treatment, with the yield improving with increased US time at fixed US power density (26.8 W/cm^2^). However, prolonging US-assisted extraction past 5 min had minimal additional benefits on the protein yield. This trend is in line with previous reports on other proteins [37,38,39]. For instance, Preece et al. [37] observed a soybean protein yield increase as the sonication time increased up to 5 min of extraction, with minimal improvement beyond this time. This behavior can be attributed to the maximum extent of protein extraction being reached for a given US power.

Protein acts as an emulsifier by creating a film over oil droplets dispersed in the solvent. Accordingly, protein can influence emulsion properties, such as creaming and coalescence. EAI and ESI are two key parameters that determine the ability of that proteins to produce and stabilize emulsions. Moreover, emulsifying properties are one of the crucial functionalities that determine the use of a protein in food manufacturing [40]. As shown in Table 2, the emulsifying properties of protein extracted with ultrasound were significantly improved compared to conventional extraction (control). In addition, EAI and ESI were enhanced as a function of sonication time up to 5 min. Greater solubility and protein structural changes attribute to the enhanced emulsifying properties, which improved protein absorption at the oil–water interface. However, further sonication caused a decrease in emulsifying properties. During alkaline extraction, most proteins are solubilized in the solvent, as the pH is away from the protein isoelectric point. With US applied during this extraction process, the hydrophilic and hydrophobic groups in protein are exposed. Prolonged US exposure may lead to more hydrophobic groups being released leading to aggregation as indicated by increased particle size from 5 min to 10 min. Similar findings were also reported for other proteins [21,41]. Therefore, a longer sonication time (10 min) resulted in a decrease in protein emulsifying properties. Based on the findings in protein yield and emulsifying properties, a sonication time of 5 min was found to be the most suitable time for conducting further experiments using the set-up in this study.

Next, the impact of ultrasound power on HPI protein yield was determined. As shown in Table 3, compared to the untreated sample, the extraction yield was increased as the US power was increased at the selected US time of 5 min. Higher sonication power can provide stronger turbulent flow, which increases mass transfer, thereby achieving a greater extraction yield. The extraction yields gradually plateaued when the ultrasound power intensity increased from 26.8 W/cm^2^ to 50.0 W/cm^2^. The maximum yield obtained under high-power ultrasound increased approximately twofold from untreated extraction. A similar trend was reported by Zhu et al. [42], where perilla seed protein yield was observed to plateau as the sonication power was increased from 60 W to 80 W. This could be due to the maximum extractability of protein being already reached at a certain power level/ultrasonication time.

Interestingly, we discovered that a significant increase of the free -SH content in US-assisted HPI samples (Table 1). The observed increase in free -SH content could be due to buried -SH groups becoming exposed to the surface of the protein following ultrasonication. This can also be supported by the decreased particle size of the extracted protein aggregates (Table 2). The particle size was significantly reduced after US treatment due to the high shear force and high turbulent flow generated in the system. Hence, it is highly possible for the -SH group to get exposed during the sonication process via the opening of the structure of individual proteins. Surprisingly, the previous investigation suggested that HIU (20 kHz, 600 W, 30 min) could break the disulfide bonds in the SPI system [18]. In contrast, the mild power and shorter sonication time used in the current study cannot cleave covalent disulfide bonds (7.1 kJ/mol). In addition, the free -SH content and the particle size were increased when the sonication power was further increased to 50 W/cm^2^. The data suggest that HIU can rapidly break apart aggregates, which exposes free -SH moieties that can eventually react with each other to form new aggregates. Interestingly, Jiang et al. [43] also observed that the free -SH content of pea protein isolate (PPI) decreased after US treatment. The difference between our study and theirs might be due to the different protein and extraction conditions (they used pH 12.0 compared to pH 10.0 in our study). The formation of disulfide bonds can account for the decrease in free -SH content, with the thiol (-SH) group tending to be a more reactive thiolate (S^−^).

### 3.3. The Impact of Ultrasound Power on HPI Structural Properties

**Figure 2:**
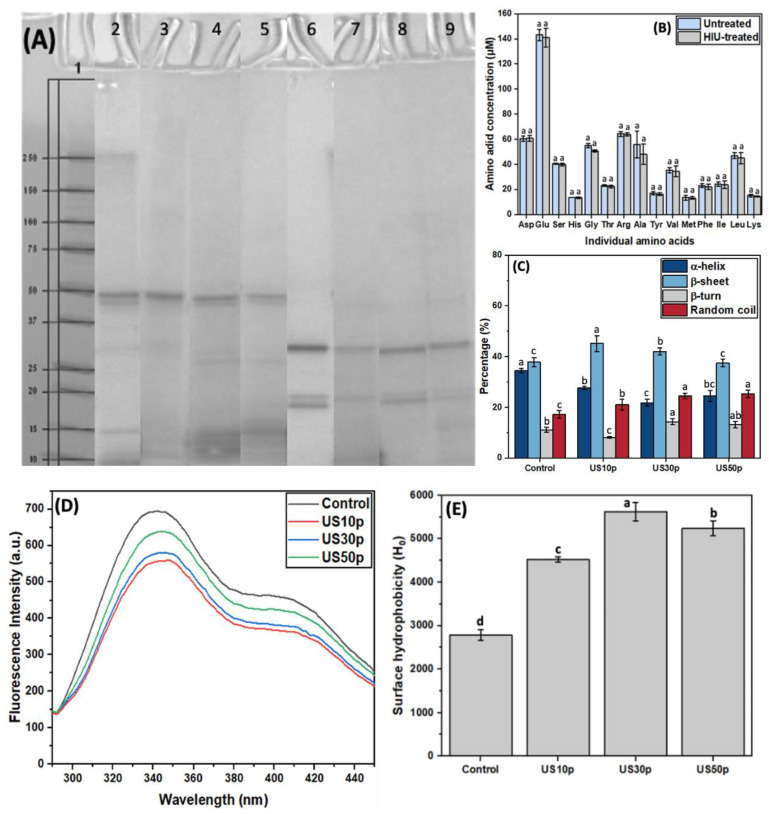
(**A**) SDS-PAGE profiles of control HPI and US-treated HPI with (reducing) or without (non-reducing) the addition of β-ME. Lanes in SDS-PAGE profiles: Lane 1: Marker; Lane 2: Conventionally extracted HPI; Lane 3: 11.2 W/cm^2^, 5 min US; Lane 4: 26.8 W/cm^2^, 5 min US; Lane 5: 50.0 W/cm^2^, 5 min US. Lanes 2–5 are under non-reducing conditions, whereas lanes 6–9 are their corresponding reducing SDS-PAGE profiles; (**B**) The amino acid profiles of untreated HPI and HIU-treated HPI; (**C**) The secondary structure of untreated and US-treated HPI under different power densities (11.2, 26.8, and 50.0 W/cm^2^ for 5 min); (**D**) Fluorescence spectra indicating tertiary structural differences between conventionally extracted and US-treated HPI under different power densities (11.2, 26.8, and 50.0 W/cm^2^ for 5 min); (**E**) Changes in surface hydrophobicity (H_0_) of conventionally and high-intensity ultrasound extracted (11.2, 26.8, and 50.0 W/cm^2^ for 5 min) HPI. The experiments were conducted in triplicate. Different lowercase letters in (**B**,**C**,**E**) represent the significant differences among different US treatments (*p* < 0.05).

Generally, the cavitation effects from low-frequency ultrasound generates both physical and chemical effects [44]. To clarify whether the generated sonochemical effects can alter the chemistry of the primary structure of HPI, the amino acid composition of the proteins in untreated and sonicated HPI were compared first. As shown in Figure S1 and Figure 2B, the major amino acid in HPI is glutamic acid (Glu), which accounted for ~20% of the overall amino acid content. Compared with untreated HPI, the amino acid profiles of the HPI suspensions were not significantly changed by exposure to ultrasound at 26.8 W/cm^2^ for 10 min. This result confirms that sonochemical effects did not chemically change any of the amino acids comprising the HPI [45], indicating that this is not a contributing factor to the modified functional properties of HPI resulting from ultrasonication.

SDS-PAGE was carried out to demonstrate the impact of ultrasound on protein integrity, while non-reducing SDS-PAGE probed interactions between protein molecules. As visualized in Figure 2A, the SDS-PAGE profile of untreated HPI (lane 2 and lane 6) was consistent with previous studies [46,47,48]. Edestin (~50.0 kDa) was shown to be the major component in HPI, accounting for more than 70% of the total protein content according to the previous study [49]. In addition, edestin is a hexameric legumin, and each monomer consists of an acidic (AS) and a basic subunit (BS). Therefore, the bands at 34.0 kDa and 20.0 kDa under reducing conditions (lane 6) correspond to edestin AS and BS, respectively. Notably, the smearing was observed on the high MW area or even stacked on the top of the gel in Lane 2, whereas it disappeared in its reducing protein profile (lane 6). This discovery confirmed that intermolecular disulfide bonds account for protein “aggregation” formation. Moreover, we did not observe the apparent smearing in other US-treated non-reducing profiles (lanes 3, 4, 5). These findings suggested a shorter peptide chain formed during sonication, leading to smaller aggregates formation. Apart from this, there were no significant changes in the non-reducing and reducing SDS-PAGE profiles of US-treated HPIs. This indicated that the ultrasound process did not significantly impact the protein subunits. In combination with the amino acids results (Figure 1), it can be concluded that the primary structure of the HPI proteins was not affected by ultrasonication. This finding was consistent with previous studies in mustard meal [50], whey protein isolate [51], and oat protein [52]. Therefore, further investigations should aim at protein secondary and tertiary structures to determine the ultrasound influences on protein structure.

In this study, we chose circular dichroism (CD) spectroscopy to evaluate the secondary structure changes, including α-helix, β-turn, β-sheet, and random coil in the ultrasound-treated HPI. The CD spectrum of untreated HPI exhibited two negative bands at approximately 208 and 222 nm and a positive band at approximately 195 nm (Figure S2). It has been suggested that these bands are an indication of the presence of α-helix [53]. Ultrasonication contributed to the negative peaks at 208 and 222 nm shifting upward and the positive peaks at 195 nm shifting downward, indicating the loss of α-helix structure in sonicated HPI [54]. In fact, compared to the untreated HPI, all samples after sonication showed a decrease in the α-helix proportion and an increase in the β-sheet and random coil (Figure 2C). Moreover, such changes in the secondary structure of HPI were power density-dependent, and higher effects were observed at medium power density levels. For example, sonicated HPI at 26.8 W/cm^2^ had a greater decrease in the α-helix than sonicated HPI at 11.2 W/cm^2^ and 50.0 W/cm^2^. Similarly, Malik et al. [55] found that ultrasound can increase the β-sheet proportion and reduce the α-helix structure of proteins. This may be explained by the fact that HPI molecules were unfolded during ultrasound treatment, leading to the disruptions of the α-helix and conversion to the β-sheet.

The fluorescence spectra of conventionally extracted HPI and US-treated HPI were obtained to determine the protein tertiary structure changes, based on the intrinsic fluorescence of tryptophan (Trp) and tyrosine (Tyr) residues, which have a high fluorescence quantum yield in hempseed protein. The other amino acids only exhibit either no or faint fluorescence. Most commonly, the protein tertiary structure changes can be evaluated by the fluorescence intensity (FI) and the maximum wavelength (λ_max_). As shown in Figure 2D, ultrasound did not significantly impact the fluorescence spectrum, but the FI and λ_max_ changed. As previously reported, λ_max_ < 330 nm indicated a non-polar adjacent environment, whereas λ_max_ > 330 nm suggested Trp residues are located in the polar environment [56]. Compared to untreated HPI, samples after sonication observed a redshift of λ_max_, indicating that the local environment’s polarity increased. This could be attributed to the embedded Trp residues exposed to the protein surface. Therefore, ultrasound can rupture the hydrophobic interactions in the protein molecules, which leads to more buried hydrophobic regions getting exposed to the protein surface. Moreover, FI showed a significant increase at 50.0 W/cm^2^ power density, which was consistent with some reported studies [19,57]. As reported, the FI intensity is proportional to the number of hydrophobic Trp residues [58]. The excessive exposure of hydrophobic regions under high US power density accounts for the increase in FI. Hence, leading to the re-formation of insoluble protein aggregates.

Surface hydrophobicity (H_0_) is an index of the number of hydrophobic groups exposed on the protein surface in contact with water. It is a crucial structural characteristic associated with protein functionality. Sonication can significantly increase the surface hydrophobicity of proteins, including pea protein isolate [59], perilla protein isolate [60], and whey protein isolate [61]. This increase could be ascribed to the partial unfolding of the protein by US turbulence flow and high shear, leading to more embedded hydrophobic regions becoming exposed. The impact of ultrasound extraction on protein surface hydrophobicity is shown in Figure 2E. The surface hydrophobicity steadily increased when the US power was increased up to 26.8 W/cm^2^, to a maximum of an approximate twofold increase over the conventionally extracted sample. This indicates that US can exert significant effects on the H_0_ of HPI, promoting more buried hydrophobic groups exposed to the polar environment by stronger cavitation effects. However, excessive hydrophobic group exposure reinforced the connection between protein molecules by hydrophobic interaction. Hence, a decline in HPI surface hydrophobicity under higher US power (50.0 W/cm^2^). Results found in surface hydrophobicity were in accord with our findings in protein tertiary structure characterization.

### 3.4. The Impact of Ultrasound on HPI Functional Properties

The significant changes in the secondary and tertiary structure and surface hydrophobicity of the ultrasonically extracted HPI suggest that functionality properties would also be altered. The main section investigated the functional properties of HPI in relation to ultrasonic treatment and the observed protein structural differences.

**Figure 3:**
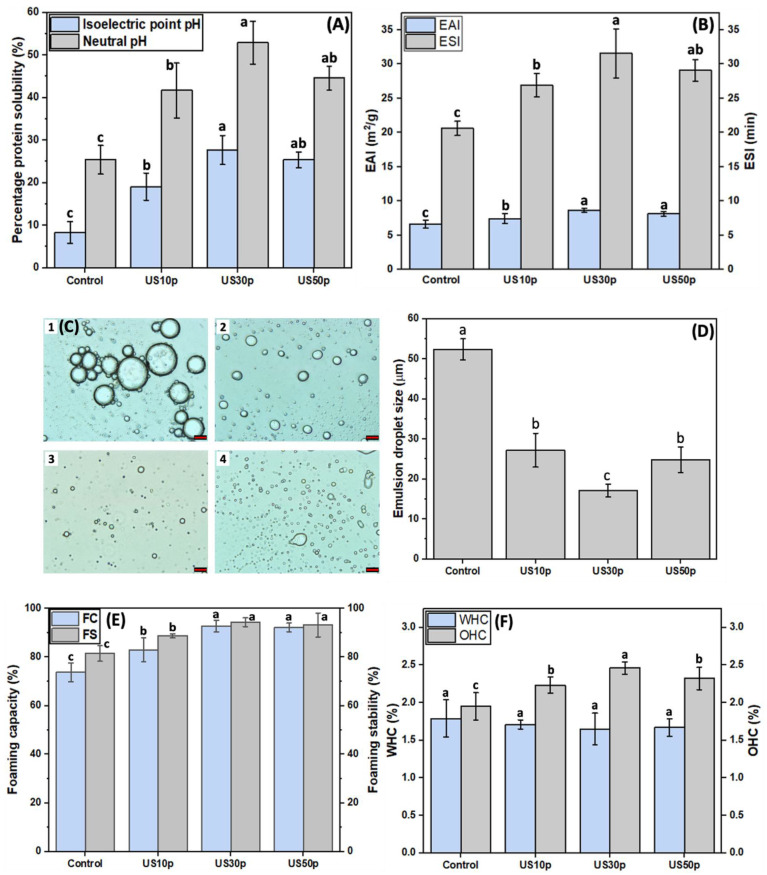
(**A**) Percentage protein solubility of untreated HPI and HPI extracted with the US-assisted method (under 11.2, 26.8, and 50.0 W/cm^2^ for 5 min) at pH 5.0 and 7.0; (**B**) Emulsifying ability index (EAI) and emulsifying stability index (ESI) of conventionally extracted HPI and HPI extracted with the US-assisted method (under 11.2, 26.8, and 50.0 W/cm^2^ for 5 min) at pH 7.0; (**C**) The microstructures of (1) Conventionally extracted HPI, (2) US10p-HPI, (3) US30p-HPI, and (4) US50p-HPI-stabilized emulsions, the scale bar in the OM is 20 μm; (**D**) The hempseed protein-stabilized emulsion droplet size; (**E**) The effect of high-intensity ultrasound extraction (11.2, 26.8, and 50.0 W/cm^2^ for 5 min) on foaming properties of HPI; (**F**) The impact of high-intensity ultrasound extraction (11.2, 26.8, and 50.0 W/cm^2^ for 5 min) on WHC and OHC of HPI. Different lowercase letters in (**A**,**B**,**D**–**F**) represent the significant differences among different US treatments (*p* < 0.05).

Protein solubility is an essential property that impacts many functional properties, such as foaming, gelling, and emulsifying capacity [62]. In particular, it is a prerequisite for protein to be further used in food applications. In general, commercial protein food applications involve weakly acidic (pH 4.0–6.0) and neutral (pH 7.0) food matrices [63]. According to the literature, HPI exhibits a typical U-shape pH-solubility profile, with the lowest solubility of only 5% at its isoelectric point (pH 5.0) [46]. Therefore, this study aims to evaluate to what extent that ultrasound can improve solubility at the common food processing pH (pH 5.0) and neutral pH (pH 7.0). As can be seen in Figure 3A, compared with the control sample, the protein solubility at pH 7.0 was significantly enhanced from ~25% to ~42% when extracted using 11.2 W/cm^2^ US. Moreover, the solubility at pH 7.0 was further improved to ~53% as the power intensity increased to 26.8 W/cm^2^. As for the solubility at pH 5.0 (isoelectric point), the maximum solubility of 27.7% was also obtained under mild power (26.8 W/cm^2^). The increased solubility could be attributed to the physical disruption of aggregates that decrease the particle size to the extent that they are registered as ‘soluble’ in the assay used. However, the solubility of HPI reduced as the power density further increased to 50.0 W/cm^2^, consistent with the increased particle size. This is in general agreement with previous investigations. Jiang et al. [19] reported that black bean protein solubility decreased under 450 W US conditions compared to mild US power (300 W). Similarly, Xue et al. [64] observed a dramatic solubility decrease in plum seed protein isolates under 158.8 W/cm^2^ in comparison to those treated at 105.9 W/cm^2^. The insoluble protein aggregates re-formation accounts for the decreased solubility. This can be evidenced by the observed secondary and tertiary structural changes.

As shown in Figure 3B, ultrasonic extraction significantly improved the emulsifying properties of HPI compared to the conventional AE-IEP. This is presumably due to the protein conformational changes caused by HIU treatment that enable more protein to engage in emulsion interface adsorption. Both the EAI and ESI were further enhanced as the power intensity was increased to 26.8 W/cm^2^, and then dropped a little at 50.0 W/cm^2^. These trends are in line with the reductions in particle size and increased protein solubility, which result from the observed structural changes and would facilitate protein interactions at the water–oil interface to increase the EAI and ESI. In addition, more retained sulfhydryl content in US30p HPI could also account for the emulsifying improvement. Compared to the HPI obtained from the conventional AE-IEP extraction method, the improvement found in US-treated HPI suggested that ultrasound is a promising technique that could ameliorate the compact protein structure. To provide further support to the observed changes in the emulsifying properties, the microstructures of the HPI-stabilized emulsions were observed under optical microscopy, as shown in Figure 3C. It is evident that the emulsion droplet size was significantly reduced in comparison to the untreated HPI-stabilized emulsion sample. The smallest emulsion droplet size was acquired under 26.8 W/cm^2^ US treatment. This is connected to the significant reduction in the emulsion droplet size from 52.4 ± 2.6 μm to 17.1 ± 1.6 μm for emulsions made using protein extracted using increasing times of sonication (Figure 3D). The result was consistent with our findings in EAI determination, indicating that US30p HPI has the best emulsifying ability as the emulsifier among all samples.

The formation of foam involves a process of protein transfer, diffusion, and re-organization at the air–water interface. The foam formation process is highly governed by surface hydrophobicity, particle size, and protein structural flexibility. Furthermore, foaming is an important property in manufacturing different functional foods. As demonstrated in Figure 3E, HIU extraction can significantly enhance the foaming properties of HPI in comparison to conventional extraction. This observation can be ascribed to the exposure of hydrophobic regions of the proteins during sonication that occur as a consequence of the observed secondary and tertiary structure changes. Ultrasonication has previously been reported to enhance protein surface hydrophobicity, hence improving the FC and FS by increasing the protein adsorption at the interface. This can be confirmed by our findings in protein surface hydrophobicity. More hydrophobic regions exposure makes protein molecules easier to absorb at the air–water interface. In addition, the smaller particle size after US extraction would enhance protein absorption to improve the FC. This result was consistent with previous studies, where Morales et al. [65] and Wang et al. [66] also discovered that smaller particle sizes after US could lead to better foaming properties of SPI. Moreover, the reduced size can form tiny air bubbles, resulting in a more stable interface, hence increasing the FS. Moreover, the FC and FS improved with the increase in sonication power. This is because higher ultrasonication power can expose more hydrophobic regions buried inside of the protein to be exposed, leading to the improvement in FC and FS. Interestingly, the improvement in foaming properties was negligible as US power density (50.0 W/cm^2^) further increased, which could be related to the apparent reaggregation observed (Table 3). A similar trend was observed by Yang et al. [67], who reported that the foaming properties of pumpkin seed protein isolate declined under high sonication power (500 W).

WHC and OHC represent the ability of proteins to retain water or oil within a three-dimensional structure physically. These functionalities can impact the quality of food products, such as the food texture, juiciness, and mouthfeel. As observed in Figure 3F, compared to conventionally extracted HPI, the OHC was improved by approximately 25%. On the contrary, the changes in WHC were less dramatic and reversed, with a minor decline under medium (26.8 W/cm^2^) and high power (50.0 W/cm^2^) ultrasound treatment. The negative correlation observed between WHC and OHC in this study was not contradictory. As aforementioned, HIU treatment can increase the exposure of hydrophobic regions on the protein surface. Therefore, the ability of protein molecules to entrap the oil is enhanced due to the affinity of hydrophobic groups to oil. In contrast, the increase in surface hydrophobicity would cause a low level of WHC. Malik and Saini [68] observed a decline in WHC and increased protein surface hydrophobicity. In the investigation of Resendiz-Vazquez et al. [69], a similar trend as shown in this study was discovered. They concluded that the negative correlation between WHC and OHC was due to the partial denaturation of the protein and the hydrophobic regions exposure after the ultrasound process, consistent with the changes in secondary and tertiary structure observed in this study.

### 3.5. Proposed Mechanism of US-Assisted Hempseed Protein Extraction System

**Figure 4:**
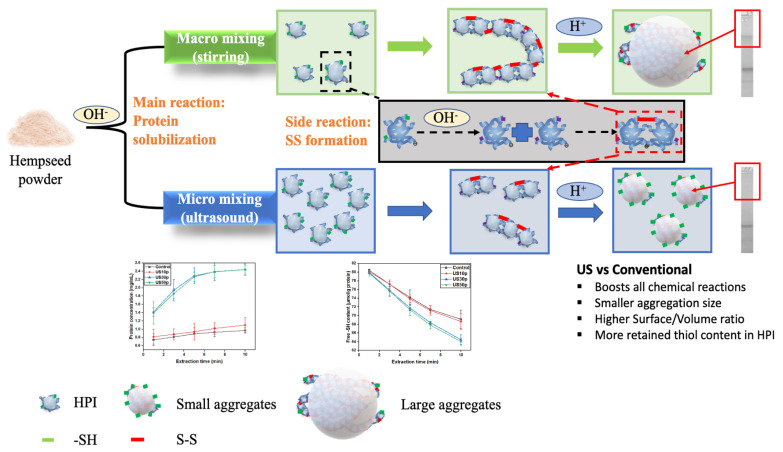
The schematic mechanism of the conventionally extracted and US-assisted extracted hempseed protein system.

As shown in Figure 4, two reactions occur simultaneously in the US-assisted hempseed protein extraction system. The protein solubilization is the main reaction, whereas intermolecular disulfide bond formation is the side reaction. Ultrasound, as the micro mixing technique, can boost all chemical processes in the system. Therefore, the protein concentration was increased, and the free -SH content of the supernatant declined. This finding is mainly because the hempseed protein can be considered fully solubilized at the alkaline condition and behaves as a peptide chain. Therefore, the thiol content measured in the supernatant is roughly equal to all thiol content in the protein, as the structure is entirely DTNB detect-accessible. However, after isoelectric point precipitation, protein gradually forms insoluble aggregates. The aggregates formed in the US-treated sample were smaller than that of the conventionally extracted sample due to the physical effects generated by ultrasound. The increased thiol content results found in US-treated HPI samples were not contradictory. Owing to the compact structure (aggregation) formed in HPI, more thiol groups were buried inside the protein. However, smaller aggregates were formed in US-treated HPI samples, and more thiols were able to bind with DTNB chemicals. In other words, more thiols exist in the initial untreated hempseed protein supernatant. However, more thiols were retained in US-treated HPI products, which is more promising for further food applications.

## 4. Conclusions

In this current research, the impact of ultrasound-assisted extraction on the structural and functional properties of HPI was comprehensively studied. In general, ultrasound-assisted extraction significantly improved the HPI yield. Compared to HPI extracted based on the conventional approach, a maximum of a 2.1-fold increase in protein yield can be achieved. The increased yield was ascribed to high shear and mass transfer effects generated by the acoustic cavitation. At neutral pH (7.0), the highest solubility of 53% obtained under 26.8 W/cm^2^ for 5 min US was about twofold that of the conventionally extracted HPI. The improvement was due to the smaller particle size of protein aggregates and partial protein unfolding under sonication treatment. In this study, ultrasound was found not significantly to impact protein subunits or amino acid compositions. Instead, alterations to the secondary and tertiary protein structure were observed, along with the exposure of hydrophobic regions. In particular, ultrasound was found to reduce the α-helix and increase the β-sheet structure of the extracted HPI, with the tertiary structure changes indicated by a fluorescence intensity decline and a redshift. Importantly, ultrasound extraction was also shown to have beneficial effects on HPI functional properties, including emulsifying properties, foaming properties, and oil-holding capacity. Notably, excessive ultrasound power (50.0 W/cm^2^) can adversely impact the functional properties of HPI due to protein aggregates re-formation. In addition, more thiol groups were retained in the US-treated HPI, which could be another reason for the functionality improvement. Overall, ultrasound is a promising technique to improve the extraction yield and broaden its potential usefulness, hence increasing the application of HPI in the food area.

## Figures and Tables

**Table 1:** The injection program for online derivatization of amino acids.

Function	Amount (μL)	Reagent
Draw	2.5	Borate buffer
Draw	1.0	Sample
Mix	3.5	-
Draw	0.5	OPA
Mix	4.0	-
Draw	0.4	FMOC
Mix	4.4	-
Draw	32.0	Injection diluent
Mix	20.0	-
Inject	2.0	-

**Table 2:** The impact of ultrasonic time (at fixed US power density = 26.8 W/cm^2^) on the protein extraction yield, particle size, and selected functionality (emulsifying properties) of HPI.

Treatment	Extraction Yield (%)	EAI (m^2^/g)	ESI (min)	Particle Size of Extracted Protein Aggregates (μm)
Control	17.6 ± 1.4 ^c^	6.6 ± 0.6 ^c^	20.6 ± 1.0 ^c^	45.7 ± 6.8 ^a^
US30p, 1 min	24.8 ± 3.6 ^b^	7.8 ± 1.2 ^b^	27.4 ± 3.3 ^b^	11.3 ± 2.1 ^b^
US30p, 5 min	35.1 ± 3.3 ^a^	8.7 ± 0.3 ^a^	31.6 ± 2.4 ^a^	5.4 ± 0.2 ^c^
US30p, 10 min	35.8 ± 2.5 ^a^	8.2 ± 0.7 ^b^	29.8 ± 1.6 ^ab^	7.2 ± 1.6 ^bc^

Note: Note: Fisher’s exact test was adopted to determine the statistical significance; ‘^a^’ represents highest average, and means denoted by ^b^, ^c^, indicate significant differences across columns at a probability level, *p* < 0.05.

**Table 3:** The impact of ultrasonic power (after 5 min sonication) on the protein extraction yield, particle size, and free -SH content of HPI.

Treatment	Ultrasound Power Intensity (W/cm^2^)	Extraction Yield (%)	Free -SH Content (μmol/g)	Particle Size of Extracted Protein Aggregates (μm)
Control	0	17.5 ± 1.6 ^b^	19.1 ± 2.1 ^c^	45.7 ± 6.8 ^a^
US10p, 5 min	11.2	19.9 ± 2.4 ^b^	32.7 ± 2.8 ^b^	19.2 ± 2.3 ^b^
US30p, 5 min	26.8	35.1 ± 3.3 ^a^	48.0 ± 4.1 ^a^	5.4 ± 0.2 ^d^
US50p, 5 min	50.0	37.3 ± 2.1 ^a^	46.6 ± 5.3 ^a^	8.9 ± 0.4 ^c^

Note: Fisher’s exact test was adopted to determine the statistical significance; ‘^a^’ represents highest average, and means denoted by ^b^, ^c^, ^d^ indicate significant differences across columns at a probability level, *p* < 0.05.

## Data Availability

The data showed in this study are available in this article.

## Supplementary Materials

The following supporting information can be downloaded at: https://www.mdpi.com/article/10.3390/foods12020348/s1. Figure S1: The amino acid profile of conventionally extracted and US-assisted extracted HPI. Figure S2: The Circular dichroism spectra of conventionally extracted and HIU-assisted extracted HPI.
