# Supplementary material for: The Impact of High-Intensity Ultrasound-Assisted Extraction on the Structural and Functional Properties of Hempseed Protein Isolate (HPI)
Source: Foods. 2023 Jan 11;12(2):348. doi: 10.3390/foods12020348 (PMC9858475; doi:10.3390/foods12020348)
Supplement: Supplementary file 1 [file foods-12-00348-s001.zip › foods-2138154-supplementary.pdf]

Supplementary Materials

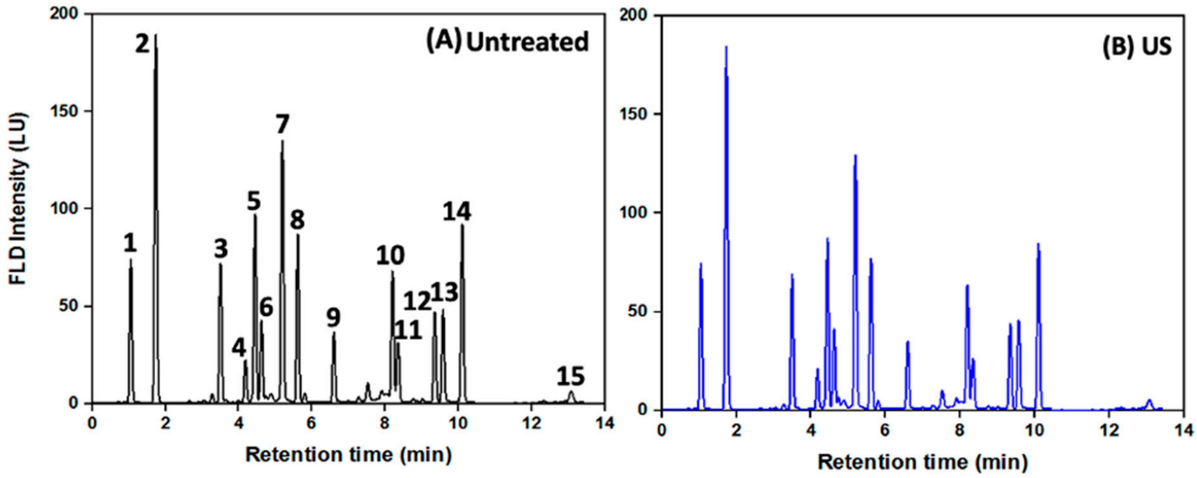

Figure S1. (**A**) The amino acid profile of conventionally extracted HPI sample; (**B**) The amino acid profile of HIU-treated HPI sample.

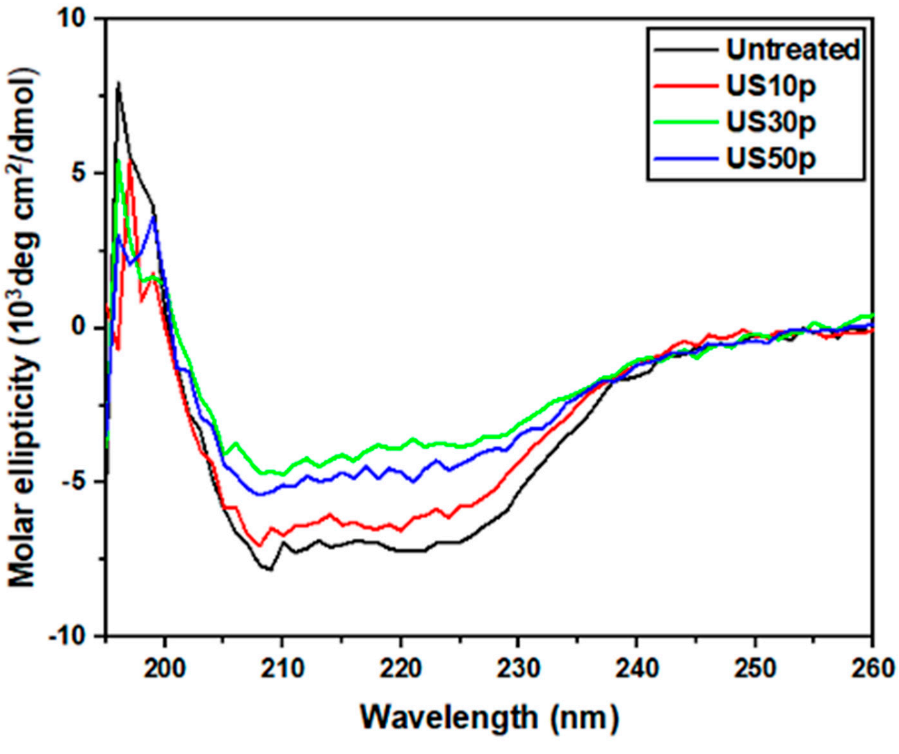

Figure S2. The Circular dichroism spectra of conventionally extracted and HIU-assisted extracted HPI.
